# Supplementary material for: Effective communication and collaboration with health professionals: A qualitative study of primary care pharmacists in Western Australia
Source: PLoS One. 2020 Jun 11;15(6):e0234580. doi: 10.1371/journal.pone.0234580 (PMC7289401; doi:10.1371/journal.pone.0234580)
Supplement: S1 File — (PDF) [file pone.0234580.s001.pdf]

Pharmacist Interview Guide

(Please note this is a guide only)

Opening Remarks:

"Thank you for your time. *The purpose of this interview is to gauge your opinion about extended and enhanced services provided by pharmacists. This will help us to make recommendations and enable strategies to be developed to improve remuneration for these services and where appropriate increase uptake of these services.*

We really value your input and honest contribution. Anything you tell us will be treated with strictest confidence and your identity will be protected.

If you have any questions regarding this research please do not hesitate to contact research team members.

The interview should take about 45 minutes. Do you consent to the interview being recorded?"

1. Where did you obtain your initial pharmacy qualification?

2. In which year did you first obtain your registration to practise as a pharmacist in Australia?

3. What is your highest qualification in pharmacy?

4. How many years have you worked as a pharmacist?

5. In one or two minutes, please describe your experiences in pharmacy.

Interviewer: *We are specifically interested in exploring enhanced and extended services provided by pharmacists. Therefore to set the scene I will read the definitions of these services to you*:

Enhanced services *are services implemented in pharmacies that are additional to or not routinely provided with prescribed or non-prescribed medicines. Examples include MedsCheck, dose administration aids service, opioid substitution program, diabetes care and weight management.*

Extended services *are defined as an extension of the existing scope of practice requiring additional education and training and a demonstration of competence to obtain additional credentialing qualifications. Extended services include medication management reviews (in patients' homes or in residential aged care facilities) and pharmacist immunisation services.*

6. Have you done any specific training related to enhanced or extended services? Do you have any other graduate and postgraduate qualifications (e.g. MBA)?

7. What are your current roles in pharmacy? If you have multiple roles, please specify these roles eg pharmacist working in community pharmacy, as an accredited pharmacist, diabetes educator, etc.

8. Please describe your current work pattern (eg 2 days per week as a community pharmacist and 3 days conducting medication reviews or different roles in a community pharmacy eg vaccination pharmacist, dispensing pharmacist, professional pharmacist)

9. What is the size of the pharmacy where you currently work? As a guide, consider:
   a. Small (approximate gross turnover < $ 1.5 million per annum)
   b. Medium (approximate gross turnover $ 1.5 – 3.5 million per annum)
   c. Large (approximate gross turnover > $ 3.5 million per annum)

10. What is the location of the pharmacy? Is it in or close to a medical centre? How far is it from the centre? What is your interaction with the med centre(s). Are there any barriers or enablers/facilitators to this?

11. Please describe the pharmacist staffing situation in the pharmacy.

12. With respect to enhanced and extended services that you offer
    a. Which ones do you think have been the most successful?
    b. Which ones do you think have been the least successful?

13. What level of research did you do before you established the most successful service and the least successful service?

Specific services:

14. What is a unique service offered in the pharmacy? Eg a service that differentiates your pharmacy from other pharmacies.
    a. What investigation did you do to implement the service eg ensuring the service was needed in your area?
    b. What processes and procedures did you have to implement eg. set-up requirements, staff training etc?

15. What were the facilitators (ie what worked) and what were the barriers (what did not work or had to be overcome) with respect to the implementation of the service?

16. Was it worthwhile implementing the service? What was the impact of offering these services in the pharmacy (eg did it generate more footprint)?
    a. Do you charge for the provision for these services? If you charge, how did you come up with this amount?
    b. If you do not charge, what impact would remuneration have on delivering this service?

17. Have you implemented a service that has had poor uptake or has not worked? Why do you think this was the case?

All extended and enhanced services:

18. What processes did you have to set up in the pharmacy to be able to offer enhanced or extended services?

19. Can you explain how the pharmacists in the pharmacy are equipped and trained (for all services or only specific services)?

20. What is the nature/ form of the collaborations with other health professionals?

    a. What do you do with respect to the collaborative interactions/ communications eg.do you have meetings once a month where the pharmacist goes to the doctor and talks to them about something eg scripts, new meds, etc? Or what do you do?

21. What do you understand ‘referral’ to mean? What do you think constitutes a referral from a pharmacist to another health professional?

22. There may be more than one way to refer a patient. Describe what process you follow.

23. Please explain referral frequencies with regard to the various services you offer.

24. With respect to your role as an accredited pharmacist, how did you know that this service was needed?

25. What requirements were there to implement the service?

26. How are referrals obtained for the service?

27. After you have interviewed the patient, have you discussed or recommended referral of any patients to another health professional with the GP?

28. What processes do you have set up to offer the service?

29. In reflecting on the service, was it worthwhile implementing the service? What was the impact of offering the services? What worked well (facilitators) and what did not work (barriers) with respect to the service? Were there any challenges?

30. Are you planning to continue providing the service? Why/ why not?

31. What is the nature/ form of the collaborations with other health professionals? What do you do with respect to the collaborative interactions/ communications?

32. After you have interviewed the patient and sent a letter of recommendations, do you get any feedback from the GP about the recommendations?

33. What is your role? With respect to this role, how did you know that this service was needed? What investigation did you have to do to implement the service eg ensuring the service would be financially viable, set-up requirements, etc

34. What requirements were there to implement the service?

35. Are there referrals for the service?

36. In the case of referrals, after you have assessed the patient, have you referred any patients to another health professional?

37. What processes do you have set up to offer the service?

38. In reflecting on the service, was it worthwhile implementing the service?

39. Are you planning to continue providing the service? Why/ Why not?

40. What is the nature/ form of the collaborations with other health professionals?

41. What is your perspective on electronic health records? Do you see this initiative as enhancing or creating barriers for the provision of professional services within the pharmacy?

42. Medicare Locals have been replaced by the Primary Health Network (PHN). The WA Primary Health Alliance (WAPHA) governs the PHN and oversees the three PHNs in WA. WAPHA encourages interprofessional collaboration.
    a. Have you heard about the PHNs and have you worked with the PHN in your area?
    b. Are pharmacists involved with the local PHN and do they attend meetings?

Thank you for taking the time to participate in this interview
