# Supplementary material for: Effective communication and collaboration with health professionals: A qualitative study of primary care pharmacists in Western Australia
Source: PLoS One. 2020 Jun 11;15(6):e0234580. doi: 10.1371/journal.pone.0234580 (PMC7289401; doi:10.1371/journal.pone.0234580)
Supplement: Attachment — Submitted filename: Response to Reviewers_PONE-D-20-04098_FINAL.docx [file pone.0234580.s002.docx]

**Manuscript ID: PONE-D-20-04098**

**Title: Effective communication and collaboration with health professionals: A qualitative study of pharmacists in the context of a changing practice landscape**

**Responses to Editor’s and Reviewers’ Comments:**

The authors would like to thank the editor and reviewers for reviewing the manuscript. We have considered all comments/suggestions and made necessary changes to improve the manuscript. Comments/issues raised by the editor/reviewers are in **BLACK**, responses to the comments are in **BLUE**, quotations from the revised manuscript are in ***RED***.

|  | **Reviewer 1’s Comments** | **Authors’ Responses** |
| --- | --- | --- |
| 1.1 | The title should be more specific to help it be identified in data base searches. For example it should include that the study is in community pharmacists, in western Australia and that one of the main findings was that there is a need for a digital health record. | Thank you to Reviewer 1 for the recommendations. Following recommendation from Reviewer 1, the full title of this manuscript has been amended as below:  “*Effective communication and collaboration with health professionals: A qualitative study of primary care pharmacists in Western Australia*”.  The term “primary care pharmacists” was used instead of “community pharmacists” as it more accurately reflects the characteristics of the participants and the intent of the study. This study included pharmacists practising in primary care settings, including in community pharmacies, independent consultant pharmacists conducting medication reviews and in residential aged care facilities. “Western Australia” was added to reflect the location of the study. Due to character limitation in the full title and for completeness, “digital health record” is now added as one of the keywords to assist with identification in database searches. The keywords are:  *“communication; collaboration; pharmacist; general practitioner; health professional; digital health record”* |
| 1.2 | The language in the introduction could be improved. At the moment it is quite verbose and could be more direct. Additionally the grammar and syntax should be reviewed (see specific comments below). Try to avoid repetitive statements too e.g. Ling 299 ’It was notable’ is repeated above. It can be removed so that the sentence reads ‘Participants had….’ | The Introduction section has been carefully reviewed and amended to make it more succinct. Necessary corrections in relation to grammar and syntax have also been made.  This line has also been amended to read “*Participants had…*” |
| 1.3 | The conclusion in the abstract is not substantiated by the results presented. e.g. ‘across the continuum of patient care’ but the study was only done in primary care, so conclusions should not be drawn across the whole care spectrum. | This sentence in the conclusion of the abstract has now been changed to:  *“The establishment of a consistent and structured two-way referral process between health professionals within the primary care setting is important, which includes use of the national digital health record.”* |
| 1.4 | The title and first paragraph refer to global contexts but the rest of the paper is quite specifically relation to Australian pharmacy practice. Consider making this explicit throughout and changing the title or update the content to include other policies, e.g. Summary Care Record as a digital health record in the UK. | The title has been amended to include “Western Australia” as per comment number 1.1. Following this advice, the Introduction section has also been amended to make it explicit for the Australian setting for consistency and improved flow. |
| 1.5 | The introduction could be improved by describing current knowledge of inter-professional collaboration and communication. There are some references to models currently available that should be introduced, e.g. https://bmjopen.bmj.com/content/bmjopen/6/3/e010488.full.pdf ; https://www.ncbi.nlm.nih.gov/pmc/articles/PMC4803863/ and https://link.springer.com/article/10.1007/s11096-017-0450-6 . The studies referred to in the discussion should be described in the introduciton. It could be stated that these models do not look at diverse enough groups of healthcare professionals (e.g. including nurses and psychologists) and so this would help the reader understand the aims of the study is too look at inter professional communication more broadly. | Currently available models as suggested by Reviewer 1 are now introduced in the Introduction section to provide a background description of current knowledge of inter-professional collaboration and communication. Further commentaries have also been added to highlight the gaps in current literature. The new paragraph is as below:  *“A systematic review conducted by Mahdizadeh et al. reviewed frameworks and models of clinical interdisciplinary collaboration published in the literature between 1990 and 2014 [31]. Although this review focused on collaboration between nurses and medical doctors, it highlighted the roles of organisational structure, social and cultural factors in facilitating clinical collaboration between these professionals [31]. A German study explored the perceptions of inter-professional communication in relation to methods and contents of communication [32]. The study found that although differences in opinions occurred between GPs and pharmacists, communication was perceived to be crucial and that any future recommendations or models should allow flexibility for these practitioners to make adjustments to suit their unique needs [32]. An Australian study conducted between 2013 and 2014 proposed a model to facilitate collaboration between pharmacists and GPs in the context of addressing medication adherence [33]. The model highlighted elements of “shared pragmatic perspectives and trust” and “regular, face-to-face interactions” [33]. Nevertheless, there has been significant progress over the last five years in relation to digital health and the provision of cognitive professional pharmacy services which may influence interactions and communication between health professionals. Currently available models also do not address pharmacists’ interactions with a diverse group of health professionals, including GPs, nurses and other allied health professionals in a contemporary practice landscape.”* |
| 1.6 | The methods are technically sounds and clearly reported. | Noted and thank you. |
| 1.7 | The results are well documents. Table 2 may be unnecessary as data is also included in the pros. | Noted and thank you.  The authors have carefully considered the need for Table 2 and felt that Table 2 provides important examples of verbatim quotations to provide readers with context to the findings reported in the Results section. The authors have carefully checked all quotations in the table and in the text to ensure no duplication. Some relevant quotations were included in the text to aid the flow of ideas presented. |
| 1.8 | Line 85: explain wha the pharmaceutical benefits scheme is. An international audience may be unfamiliar with this terminology. | The following has been added to describe the Pharmaceutical Benefits Scheme and provide context to “price disclosure”:  *“The Pharmaceutical Benefits Scheme (PBS) has been a long-standing platform for the provision of a wide range of medicines readily accessible to the public from community pharmacies, subsidised by the Australian Government [8, 9]. Price disclosure refers to a program within the PBS which “progressively reduces the price of some PBS medicines which are subject to competition, ensuring better value for money from these medicines” [8]. This change has reduced pharmacist remuneration from the PBS, but has been coupled with a positive trend in government funding for professional services through several Community Pharmacy Agreements [10].”* |
| 1.9 | Line 88 - 91: this is a very long sentence that is quite verbose. Try to split into two sentences or make it shorter and to the point. | The sentence has now been amended and reads:  *“The increased provision of professional services by pharmacists augments the requirements to interact with and involve patients through shared decision-making. It also increases the obligation for communication and collaboration with other healthcare professionals who are involved in the care of the patients.”* |
| 1.10 | Line 93: ‘has also been’ rather than ‘has been also’ | This has now been amended to *“has also been”.* |
| 1.11 | Line 114: this paragraph could go a little further to provide some critical reflection on the introduction of My Health Record. What were the expectations? Preliminary evaluation? Lesson’s learned from other contexts? | As recommended, the following has been added to the paragraph:  *“The effectiveness and risk management of the implementation of this system under the opt-out model was evaluated by the Australian National Audit Office (ANAO) [29]. It reported the implementation was considered “effective” and “appropriate”. The report further highlighted the robust controls which were put in place to enable cyber security and privacy, although on-going regular monitoring for compliance with access and security legislation was recommended. The number of active records has increased by 190,000 since July 2019 to a total of 22.71 million. Following implementation campaigns, over 90% of community pharmacies and general practices are registered with the system [30].”* |
|  | **Reviewer 2’s Comments** | **Authors’ Responses** |
| 2.1 | Thank you for the opportunity to review this paper. I believe it touches an important topic. I have some suggestions that I believe will strengthen this paper: | Noted and thank you. |
| 2.2 | Title  - As the focus of this manuscript is primary care pharmacists – I believe this should be stated in the title. | The title of the manuscript has been amended to:  “*Effective communication and collaboration with health professionals: A qualitative study of primary care pharmacists in Western Australia*”. |
| 2.3 | Introduction  - Due to the international audience, I think it would be helpful to expand what you mean by “Ongoing medicine price disclosures and reductions which commenced in 2007, have affected the Pharmaceutical Benefits Scheme [10, 11]. These changes, coupled with a positive trend in government funding for professional services through several Community Pharmacy Agreements [12]”. Define price disclosure and professional services that are funded. To allow for this extra space, I believe you can delete lines 74-81. | Following recommendation by both Reviewers 1 and 2, the following sentence has been added to describe the Pharmaceutical Benefits Scheme and price disclosure:  *“The Pharmaceutical Benefits Scheme (PBS) has been a long-standing platform for the provision of a wide range of medicines readily accessible to the public from community pharmacies, subsidised by the Australian Government [8, 9]. Price disclosure refers to a program within the PBS which “progressively reduces the price of some PBS medicines which are subject to competition, ensuring better value for money from these medicines” [8].”*  Professional services that are funded refers to services which are remunerated through the *“Community Pharmacy Agreements”*. A reference has also been provided. |
| 2.4 | Method  - Why were only pharmacists in WA recruited? | Pharmacists in WA were recruited as this study was funded by the Pharmaceutical Society of Western Australia J M O’Hara Research Fund which aims to promote local research and advance pharmacy practice in Western Australia. However, primary care pharmacy services are similar across Australian jurisdictions and the results would therefore be transferable to other states and territories. |
| 2.5 | Can you expand on the purposive sampling. It is unclear how you prioritized some participants over others. I assume there would be hundreds of pharmacists meeting your inclusion criteria, it is not clear why some were chosen over others (e.g I am sure there are plenty of pharmacists that can administer immunization services, how did you choose which one to recruit?). | Participants were selected based on purposive sampling principles to enable the selection of information-rich cases and takes into consideration participants’ availability and ability to express an opinion in an articulate manner. Further information has been added to the Materials and Methods section of the manuscript:  *“Purposive sampling allowed maximum variation, thus enabling in-depth inquiry into a range of professional services provided by pharmacists in the current practice landscape [35]. Through the researchers’ network and consultation with pharmacy professional bodies, an initial list of prospective participants was developed. In addition to practice backgrounds and experience, participants’ willingness and availability to participate as well as articulation and communication skills were taken into consideration. Subsequent participants were recruited via the snowballing method [36]. The prospective participants were contacted and invited to participate in the study via email or telephone between September 2017 and February 2018. This purposive recruitment method enabled pharmacists with additional qualifications to provide extended services such as pharmacist-administered immunisation services, diabetes education, wound management, integrated healthcare and specialised compounding to be included. The diverse backgrounds and characteristics of participants enabled an information-rich discussion.”* |
| 2.6 | Due to the international audience, you should define accredited pharmacist (perhaps in the intro is best for this). | The following has now been added to the Introduction section to define “accredited pharmacists”:  *“Accredited pharmacists similarly have regular interactions with other health professionals. In Australia, these pharmacists are accredited with either the Australian Association of Consultant Pharmacy (AACP) [20] or the Society of Hospital Pharmacists Australia (SHPA) [21]. They conduct Australian government-funded medication reviews for patients in their home or for residents of RACFs.”* |
| 2.7 | What is an independent interviewer? I am not sure what the relationship is, but if they were paid by the research team or a student, are they independent? You state you followed the COREQ item checklist, but I do not see you report on all 8 items for the Personal characteristics of the interviewer and researchers. | Thank you for the query. The independent interviewer was engaged in this project for the sole purpose of conducting the interviews, and was not involved in the design of the study, the construction of the interview tool or data analysis. The research team had selected this interviewer as the interviewer had prior experience in conducting interviews and was a pharmacist, which enabled appropriate digression of the interview conversation for information-rich data. The delineation of this role minimised any potential bias or influence on the participants’ responses.  “Domain 1: Research team and reflexivity” of the COREQ checklist has also been carefully examined and further information added to the manuscript to report on all eight items. Below is a summary:   1. *Interviewer/facilitator:* included in the “Interview tool and data collection” section 2. *Credentials:* included in the cover page 3. *Occupation:* included in the cover page and in the section “Materials and Methods” 4. *Gender:* this information is now included for all researchers and the interviewer 5. *Experience and training:* this information is now included for all researchers and the interviewer 6. *Relationship established:* to avoid bias, there were no special relationship established or conflicts of interest with any participants prior to study commencement 7. *Participant knowledge of the interviewer/project:* this has now been added to the manuscript. All participants were provided with a participant information statement with details about the study, including the background and study objectives and contact details of the research team. 8. *Interviewer characteristics:* this information is included in the “Interview tool and data collection” section. |
| 2.8 | Line 187 mentions COM-B model for the first time. This should be described for the reader. (I am also not clear how it was used in the results) | This information was initially included in the manuscript for completeness. Data from this project in relation to factors influencing implementation and maintenance of service was reported in a separate manuscript. In light of the reviewer’s comment, to improve clarity and avoid confusion, the sentence has been amended to the following without reference to the COM-B model which was not relevant to the findings reported in this manuscript:  *“Other data relevant to factors influencing the implementation and maintenance of professional pharmacy services were reported elsewhere [6].”* |
| 2.9 | Data saturation is not talked about in the method. Please add how this was defined. | The following has now been added to the “Data analysis” section:  *“Data saturation was considered when no new codes were identified during the coding process.”* |
| 2.10 | Was the data analysed independently by the 2 researchers? | Data was analysed independently by the two researchers. The following has been added to the manuscript:  *“Two researchers (TFS and LH), both pharmacist academics with experience in qualitative research, analysed the data independently following an inductive approach to facilitate thematic analysis [36, 37].”* |
| 2.11 | How was disagreement between the 2 researchers handled? | The following has been added to the manuscript to clarify how disagreement was handled between the two researchers:  *“During the data analysis phase, the two researchers had regular meetings to openly discuss any disagreements to reach consensus. When consensus was not reached, the other two researchers were involved to resolve any disagreements.”* |
| 2.12 | Results  - As this was purposive sampling, can you also add a table with the total participants demographics (not individual such as table 1). I cannot see if you have the spread that you were aiming for. E.g. if you were purposively aiming for equal gender, why is this not 50% for each gender. Same comment to see if there are equal distribution for roles, accreditation, specialized roles etc. It is hard to determine if saturation for each of these demographics was obtained if it is unclear how many pharmacists in each group participated. | Thank you for the comment. The authors have thoroughly considered this suggestion. Table 1 provides detailed description of each of the 26 participants. As per the methodology, to enable a diverse and rich collection of data, this qualitative study had intentionally recruited participants with unique and varied characteristics. As the characteristics and backgrounds of each of these participants were unique, it was not possible and would be contrary to qualitative methodology to provide a quantitative measure to these participants or the data. Following the principles of qualitative and exploratory research, despite the diverse characteristics of participants, the same interview tool was used as a guide to meet the study objectives. Data saturation was then determined based on the themes emerged through the interviews on the topics being discussed, as opposed to saturation of the demographics of participants. |
| 2.13 | For ‘Means of communication and systems’ you discuss different practitioners that pharmacists communicate with. This does not seem to fit the theme ‘Means of communication’. | The different practitioners were raised by participants as they identified and discussed various scenarios which involved interactions and communication with other health professionals. These different practitioners were coded as one of the ‘codes’ which was later decided to be reported under the theme “means of communication and systems” as it relates to the theme about needing a range of distinctive methods of communicating with individual practitioners. The reporting of the types of practitioners in this section provided context to the aforementioned theme. However, amendments have now been made to this section to improve the link between these two codes:  *“During the interviews, participants described a range of distinctive methods of communicating with various health professionals, and the importance of developing and tailoring a method of communication that worked for both parties. It was important to note that the communication method may have differed dependent on the practitioner with which the pharmacist communicated. Having an established and consistent communication system with an individual practitioner was deemed to greatly enhance collaboration as practitioners were ‘used to’ being contacted in that manner.”* |
| 2.14 | The aim of your study is to ‘explore contemporary processes facilitating communication and collaboration between community pharmacists and other health professionals.’ A means of communication alone (e.g. having a telephone) does not seem to be a contemporary process to facilitate communication. I am not sure if your themes (i) means of communication, ii) referral processes, iv) barriers to effective interactions are relevant to your aim. Theme 3 and 5 might be the main answer to your aim and might benefit from being reanalysed with a clear answer to your aim. | Although communication via a ‘phone call’ is not a ‘new’ method of interaction, it emerged from this study as one of the currently used means of communication, along with other methods, hence the report of this finding. However, it is important to note that the key finding as reported here was the variety and diverse methods of communication which needed to be tailored to the individual circumstances, including taking into consideration the practitioners being contacted and the nature and intended complexity of the interaction. This is included in the Results section as below:  *“The nature and intended complexity of the interaction was also raised as a point for consideration. For example, if the communication was intended to be brief and straight-forward, a phone call was considered the most effective way…”*  Thank you also for raising the point about clarity of our objective. The sentence about the objective of the study has been amended to better reflect the study objective:  *“This study aimed to explore contemporary processes to achieve effective communication and collaboration between community pharmacists and other health professionals, through exploring pharmacists’ views and experiences in the context of a changing practice landscape.”*  The research team have carefully revisited and reconsidered the thematic analysis addressing the objective. Upon thorough consideration, the first theme “Means of communication and systems” have been rephrased as *“Tailored means of communication”* to better reflect the key findings. All other four themes further address processes for achieving effective communication and collaboration. |
| 2.15 | Line 251 states ‘Having an established communication means or system, which enables operational efficiency, was identified to have a major impact on the success of developing and maintaining an on going positive collaborative working relationships with other health professionals’. Why is this a separate theme to ‘Facilitators to effective interactions’. This comment might be linked to my comment on your aim. | This sentence has now been amended to *“Having an established diverse communication means or system, which enables operational efficiency, was identified to have a major impact on the success of developing and maintaining an on-going positive collaborative working relationships with other health professionals”* to emphasize the importance of ‘diverse’ communication means as a process of communication, and was repeatedly raised by participants, therefore reported as a separate theme. As previously mentioned, the first theme has also been reworded as *“Tailored means of communication”* to better reflect the key findings. |
| 2.16 | ‘Means of communication’ appears to just list different ways pharmacists communicate. Qualitative analysis should add some extra depth to this. Such as when one means of communication is preferred over another, to highlight the ‘why’ not just the ‘what’ (which could be answered in a survey). Further analysis of this theme may be required to identify the nuances of different modes of communication. | Upon thorough consideration and in light of Reviewer 2’s comments, this theme has now been rephrased as *“Tailored means of communication”* to better reflect the key findings. This theme reports the important finding of having a diverse method of interaction, tailored to the individual practitioner and scenario, and takes into consideration when one method is preferred over the other based on the nature and complexity of the interaction. Further information has also been added to the manuscript to report on the ‘why’ and ‘how’ each method was preferred over the others, with appropriate supporting quotes to provide context to the narration. For example:  *“It was important to note that the communication method may have differed dependent on the practitioner with which the pharmacist communicated. Having an established and consistent communication system with an individual practitioner was deemed to greatly enhance collaboration as practitioners were ‘used to’ being contacted in that manner.”*  *“The nature and intended complexity of the interaction was also raised as a point for consideration. For example, if the communication was intended to be brief and straight-forward, a phone call was considered the most effective way…”*  *“For other interactions especially in relation to medication reviews where more complex clinical recommendations were involved, written communication with clear documentation that enabled follow up were the preferred communication means. Specifically, one participant who provided Home Medicines Reviews articulated the process of two-way written communication with the prescriber…”*  *“Where suitable, regularly scheduled face-to-face meetings were also raised by some participants as an effective way to facilitate interactions especially if the interaction was likely to involve lengthy discussion and deliberation…”* |
| 2.17 | The means of communication don’t seem particularly contemporary, as per your aim. Perhaps focus more on the contemporary processes instead of the traditional ones. | As mentioned above, this theme has been rephrased as “*Tailored* means of communication” to better reflect the key points. It was evident from this study that participants found a key contemporary process to achieve effective communication and collaboration with other health professionals was the presence of a diverse means of communication, tailored to meet the individual practitioners’ needs and the nature and complexity of the intended interaction. This has now been clarified throughout the manuscript. In addition, some pharmacists now contact a prescriber directly by phoning the prescriber’s mobile phone instead of phoning the practice. This emphasised that they had a direct and efficient/quick link to the prescriber. This has now been clarified in the manuscript. |
| 2.18 | I am not sure why ‘national digital health record’ is a separate Theme from ‘means of communication’. The national digital health record seems to be another way for practitioners to communicate. | This relates to the previous sections. As mentioned above, the first theme has been rephrased to emphasize the importance of having a diverse and tailored means of communication, instead of merely reporting on the different ways of communicating. The ‘Implementation of a national digital health record’ emerged as a theme in this study as participants described processes to achieve effective collaboration. The national digital health record is not a direct means of communication with other health professionals, but rather indirect through the uploading of patients’ health information to facilitate decision-making and collaboration. Although it is not a direct means of communication, it has been identified as an important piece of process to enable inter-professional collaboration in the management of a patient. Further information about the national digital health record system has also now been added to the Introduction section to provide context to international readers. |
| 2.19 | Discussion  - There is a large focus on ‘referral’ in your discussion. However, this does not seem particular relevant to your aim of ‘contemporary processes facilitating communication and collaboration between community pharmacists and other health professionals.’ More emphasis should be placed on these contemporary processes (Maybe you need to define what these are e.g. would instant messaging apps like WhatsApp fit this?) | Thank you for the comment. The focus on ‘referral’ in the discussion is because it was evident from this study that many participants acknowledged that with the changing landscape of the Australian health system, referring patients to another health professional (usually a general practitioner) was an effective and necessary contemporary process for communication and collaboration. In the primary care setting, communication relating to referrals has to be tailored to the individual scenario and could involve varying levels of formality, urgency and detail. However, currently, a consistent and structured two-way referral process between pharmacists and other health professionals in the primary care setting is lacking for most professional services. This important point is discussed in the Discussion section accordingly. |
| 2.20 | Line 534 states “In this study, the lack of other health professionals’ understanding and/or appreciation and respect of pharmacists’ roles and expertise was identified as one of the major barriers to effective interactions.” As you did not interview other health professionals’ I do not believe you can make this claim. | Thank you for the comment. This sentence has now been removed as recommended. |
|  | **Reviewer 3’s Comments** | **Authors’ Responses** |
| 3.1 | Thank you for the opportunity to review this manuscript. At the time I was reading this, the COVID-19 pandemic had been in full swing for several weeks. The crisis and strain, particularly on primary care health professionals, highlight the importance of this study and supports the recommendations. | Noted and thank you for your comment. The authors agree with Reviewer 3’s sentiments around the importance of the study and recommendations amid the current pandemic. |
| 3.2 | Please find below comments for your consideration.  As this is an Australian study it would be helpful to provide a paragraph about the Australian context for pharmacists – e.g. pharmacists don’t have prescribing rights, pharmacists are only just starting to work in non-prescribing roles in GP practices, there is no formal referral process in place such as is available to pharmacists in Scotland’s Minor Ailments Scheme, formal referral processes are in place for other health professionals. | Thank you for the suggestion. The following is now included in the Introduction section to provide the Australian context for pharmacists:  *“As part of the paradigm shift, defined career pathways where pharmacists are increasingly embedded within other primary care practice settings, have been established. These include working alongside other health professionals, including GPs, in residential aged care facilities (RACFs) and Aboriginal Health Services (AHSs) [13, 14]. In addition to GPs, close-working relationships between community pharmacists and other health professionals, either co-located within, or independent of a community pharmacy, have become a more common phenomenon in primary care. This includes interactions with nurse practitioners [15-17] and psychologists [18, 19]. Accredited pharmacists similarly have regular interactions with other health professionals. In Australia, these pharmacists are accredited with either the Australian Association of Consultant Pharmacy (AACP) [20] or the Society of Hospital Pharmacists Australia (SHPA) [21]. They conduct Australian government-funded medication reviews for patients in their home or for residents of RACFs. Although the Pharmacy Board of Australia supports and has declared no regulatory barriers for pharmacists to prescribe under supervision or a structured collaborative arrangement [22], pharmacists in Australia do not currently have prescribing rights resembling the models in the United Kingdom (UK) [23] and Canada [24]. There is also a lack of a formal and remunerated referral process in place for Australian community pharmacists in relation to the management of minor ailments similar to those associated with the Minor Ailments Scheme in the UK [25].”* |
| 3.3 | Line 26  Suggest change  “the primary care setting requires an augmented need to communicate and collaborate with other…”  To  “the primary care setting augments the need to communicate and collaborate with other…” | The sentence has been amended accordingly, and now reads:  *“The expanded provision of professional services by community pharmacists in the primary care setting encompasses the necessity to communicate and collaborate with other health professionals.”* |
| 3.4 | Line 39 and 235 and 353  Suggest change “facilitators to effective interactions” to “facilitators for effective interactions” or “facilitators of effective interactions” | The sentence has been amended accordingly, and now reads:  *“…facilitators for effective interactions…”* |
| 3.5 | Line 40-42  Suggest change:  “Participants acknowledged the changing landscape of the Australian health system that affects communication and collaboration with other health professionals.”  to  “Participants acknowledged that the changing landscape of the Australian health system affects communication and collaboration with other health professionals.” | The sentence has been amended accordingly, and now reads:  *“Participants acknowledged that the changing landscape of the Australian health system affected communication and collaboration with other health professionals.”* |
| 3.6 | Line 87  Change has to have | This has now been actioned and changed to “have”. |
| 3.7 | Line 89  Change would to will (if you anticipate something it is in the future)  Suggest change to “.. by pharmacists will augment the need to interact and …” | This has now been changed and the sentence now reads:  *“The increased provision of professional services by pharmacists augments the requirement to interact with and involve patients through shared decision-making.”* |
| 3.8 | Line 91  Add the word ‘to’  “healthcare professionals involved or referred to for the care of such patients” | Following recommendation from Reviewers 1 and 3, this sentence has now been rephrased to:  *“It also increases the obligation for communication and collaboration with other healthcare professionals who are involved in the care of the patients.”* |
| 3.9 | Line 93  Change  “professional pharmacy services has been also identified in previous…”  To “professional pharmacy services has also been identified in previous…” | The sentence has been amended accordingly, and now reads:  *“…professional pharmacy services has also been identified in previous Australian studies.”* |
| 3.10 | Line 120  Suggest change  “lead to systems that operate for improved interdisciplinary collaboration..”  To  “lead to systems that enhance interdisciplinary collaboration…” | The sentence has been amended accordingly, and now reads:  *“An understanding of these processes should lead to developing systems that enhance interdisciplinary collaboration and patient care.”* |
| 3.11 | Line 118  Change  “current processes leading to effective communication..”  To “current processes which lead to effective communication…” | The sentence has been amended accordingly, and now reads:  *“…little is known about current processes which lead to effective communication…”* |
| 3.12 | Line 120  Suggest change to “lead to developing systems that enhance interdisciplinary collaboration and patient care.” | The sentence has been amended accordingly, and now reads:  *“…lead to developing systems that enhance interdisciplinary collaboration and patient care.”* |
| 3.13 | Line 134  I note that not all items of the COREQ checklist have been reported. | Further information has now been added to the Materials and Methods section to carefully report all items of the COREQ checklist. |
| 3.14 | Line 147  Were community pharmacists, accredited pharmacists, and pharmacists with additional qualifications recruited? I associate accredited pharmacists as being accredited to provide HMRs and RMMRs through gaining additional qualifications – usually through AACP. Readers who are not Australian probably won’t make this connection. However I do not associate pharmacists who are accredited to immunise through additional training as accredited pharmacists.  In line 161 there appears to be a distinction between accredited pharmacists (Part D) and pharmacists with additional training (Part E). | To provide clarity for international audience, further information about accredited pharmacists has been added:  *“In Australia, these pharmacists are accredited with either the Australian Association of Consultant Pharmacy (AACP) [20] or the Society of Hospital Pharmacists Australia (SHPA) [21]. They conduct Australian government-funded medication reviews for patients in their home or for residents of RACFs.”*  Immuniser pharmacists were referred to as *“I: Immuniser pharmacist”*; whilst accredited pharmacists were referred to as *“A: Accredited pharmacist”*.  Part E referred to pharmacists with additional training (other than accreditation with the AACP or SHPA), and included those with diabetes education, wound care, specialised compounding, integrated health, and were reported in Table 1. |
| 3.15 | Line 158  It would be useful to have access to the interview question guide. | As requested, the interview guide is now provided as a Supplementary file to the manuscript. |
| 3.16 | Line 181  Was the analysis conducted by the two researchers independently or was it conducted collaboratively? | The analysis was conducted by the two researchers independently, and this has now been clarified in the manuscript. |
| 3.17 | Line 184  Change “processes to effective “ to “processes for effective” | The sentence has been amended accordingly, and now reads:  *“processes for effective”* |
| 3.18 | Line 187  What is the COM-B model? Is there a reference for this model? | This information was initially included in the manuscript for completeness. Data from this project in relation to factors influencing implementation and maintenance of service was reported in a separate manuscript. In light of the reviewer’s comment, to improve clarity and avoid confusion, the sentence has been amended to the following without reference to the COM-B model which was not relevant to the findings reported in this manuscript:  *“Other data relevant to factors influencing the implementation and maintenance of professional pharmacy services were reported elsewhere.”* |
| 3.19 | Line 244  Change “that” to “who” | This has now been actioned – “that” has been changed to “who”. |
| 3.20 | Line 253  Change “relationships” to “relationship” | This has now been actioned – “relationships” has been changed to “relationship”. |
| 3.21 | Figure 1  This figure works well to illustrate the point. | Noted and thank you. |
| 3.22 | Line 486-489  Suggest dividing into two sentences. | This long sentence has now been divided into two sentences, and now reads:  *“All 26 pharmacists in this study had unique and varied qualifications and practice experiences including some in specialist services. This further highlighted changes in the scope of pharmacist practice towards specialisation to meet contemporary needs.”* |
| 3.23 | Line 499  Add “used to”  “methods they used to manage referrals” | The sentence has been amended accordingly, and now reads:  *“…methods used to manage referrals…”* |
| 3.24 | Line 500  Change “actions” to “action” | This has now been actioned – “actions” has been changed to “action”. |
| 3.25 | Line 517  Change “referrals” to “referral” | This has now been actioned – “referrals” has been changed to “referral”. |
| 3.26 | Line 519-520  The grammar is not correct in this sentence.  I also do not see how the conclusion of GPs having power over pharmacists is derived. | Upon thorough consideration, this claim has now been removed. |
| 3.27 | Line 553  Change to  “This study identified that overall pharmacists were supportive of..” | The sentence has been amended accordingly, and now reads:  *“This study identified that overall pharmacists were supportive of the establishment and implementation of a national digital health record system…”* |
| 3.28 | Line 569  Change “personnel” to “person” | This has now been actioned – “personnel” has been changed to “person”. |
